# The radiation response measurement of a single and multiple cell ionization of neuroblastoma cells by infrared laser trap

**DOI:** 10.1093/jrr/rrac082

**Published:** 2022-12-15

**Authors:** Mulugeta S Goangul, William C Stewart, Daniel Erenso, Horace T Crogman

**Affiliations:** Department of Physics, Addis Ababa University, Addis Ababa 1000, Ethiopia; Department of Biology, Middle Tennessee State University, Murfreesboro, Tennessee 37132, USA; Department of Physics and Astronomy, Middle Tennessee State University, Murfreesboro, Tennessee 37132, USA; Department of Physics, California State University Dominguez Hills, Carson, California, 90747, USA

**Keywords:** laser trapping (LT), neuroblastoma (NB), multiple and single cell ionization, Threshold Ionization Energy (TIE), Threshold Radiation Dose (TRD), cancer, murine Neuro-2A

## Abstract

Neuroblastoma (NB) is a common type of cancer found mostly in infants and arising from the immature neural crest cells of the sympathetic nervous system. Using laser trapping (LT) technique, the present work contributes to advancing radiotherapy (RT), a leading treatment method for cancer. A single, 2-cells, 3-cells, 4-cells, and 5-cells were trapped using the high-intensity gradient infrared laser at 1064 nm and allowed to become ionized. In this work, a systematic study of Threshold Ionization Energy (TIE) and Threshold Radiation Dose (TRD) versus mass for both single and multi-cell ionization using laser trapping (LT) techniques on NB is presented. The results show that TIE increased as the mass of cells increased, meanwhile TRD decreased with the increase of cell mass. We observed an inverse correlation between TRD and cell mass. We demonstrate how to compute the maximum radiation dosage for cell death using the LT technique. Results show a possible blueprint for computing the TRD *in vivo*. The use of multiple cell ionization to determine radiation dosage along with better data accuracy concerning the tumor size and density will have profound implications for radiation dosimetry. The diminution in TRD becomes more significant in multiple cell ionization as we see in TRD vs the number of cells entering the trap. This is due to the chain effect generated by radiation and the absorption by water molecules at 1064 nm. This result provides us with better insight into the optimization of the therapeutic ratio.

## INTRODUCTION

Cancer is a leading cause of death worldwide in children [1]. Of the various types of pediatric cancers, the third most common is neuroblastoma (NB) [2–4] which arises from the immature neural crest cells of the sympathetic nervous system [5]. Almost 90% of it is found in infants not older than 5 years, and about 67% of children with NB are diagnosed late. By this time, the lymph nodes and other body parts are affected, making it very hard to cure. Various treatments such as generic medicine, surgery, radiation, or a combination are used to attack most cancers in children, nonetheless with low survival rates in the case of NB (40–50% for a 5-year survival rate [2–4]); note that these numbers vary from one country to another. Of roughly 300 000 children diagnosed with cancer each year, more than 80% are treated without recidivate in high-income countries; on the other hand, only about 20% are treated without recidivate in many marginalized countries [6, 7]. Although no precise data exist in the case of NB, the mortality rates in poor countries such as Ethiopia for most childhood cancers are nearly 100% [8].

High-risk NB is treated with induction chemotherapy, stem cell transplant, or an association of stem cell transplant with a large chemotherapy dosage before radiotherapy (RT) is administered [9]. Doctors tend to avoid RT due to the growing concern of radiation-induced tissue toxicity [10]. Radiation is most dangerous to cells during the mitosis (M) and G2 phases of the cell cycle, and they are most resistant during the late S phase [11]. Radiation damages cells by causing DNA breaks on both single- and double-stranded strands, resulting in cell death through various mechanisms such as mitotic catastrophe [12]. These breaks are repaired by nonhomologous recombination and homologous recombination. Further, as tumor cells have certain molecular vulnerabilities that normal cells do not possess, targeting alternate pathways can render the tumor radiosensitive while leaving normal tissue relatively unaffected [13]. Many signaling pathways in normal cells, including those responsible for DNA repair, exhibit redundant functions [14]. Deficiencies in p53 pathway signaling will prevent radiation-induced cancer cells from arresting at the G1/S checkpoint and will leave them vulnerable to drugs that inhibit the G2 checkpoint; they will also proceed into mitosis before they can repair their radiation-induced DNA damage [14, 15]. As a result, there is a high risk of mitotic catastrophes and cell death. Radiation can also cause replicative senescence, which impairs a cell’s ability to divide [16]. Therefore, due to the fact that cancer cells have mutations that result in the loss of redundancy, cell death can be induced by targeting the DNA damage response pathways in the cancer cells [17].

During cancer treatment, *the therapeutic ratio* measures the balance between killing cancer cells and radiotoxicity for healthy cells. This is the main factor that aids in stopping tissue toxicity caused by radiation and improving cancerous cells’ sterilizing process [10]. To obtain a favorable tradeoff between treatment benefit and morbidity, there must be a balance of radiation effectively destroying cancerous cells while preventing the detrimental effects of radiotoxicity on the healthy cells [18, 19]. To control the disease while still preserving normal tissue tolerance levels, a more precise therapeutic ratio must be established.

One step closer to determining a more optimized therapeutic ratio is through Laser trapping (LT). Therefore, in this study, we use high-power LT to better understand the therapeutic efficacies of carcinoma cells. LT is an advanced technique that utilizes radiation force to capture, translate and manipulate microscopic particles [10, 20]. This new experimental method has played a revolutionary role in the areas of physical and biological sciences. It was recently demonstrated that the ionization energy (IE) and the charge developed could be determined at the single-cell level for BT20 breast cancer [19] and RBCs cells [18, 20, 21]. Further, in a high-power trap (50 mW), the blood cells undergo hemolysis as they become charged, and are ejected, which is not the case in a low-power trap. In another study using LT for ionization of multiple 4 T1 breast carcinoma cells, a significant reduction in the Threshold Radiation Dose (TRD) was observed due to ionization by chain interaction [22].

The main idea of this study is that the laser trap can be used to measure Threshold Ionization Energy (TIE) and TRD as a function of the mass of single and multiple NB cells. This type of approach has the potential to increase our understanding of how responsive cancer cells are, to specific energy levels and dosages which directly improves the accuracy of RT. We present here a systematic study of TIE and TRD for both single and multi-cell ionization processes, using LT techniques. Further, we will examine and compare whether the effect found in the 4 T1 breast cancer cell lines [22] can be confirmed in other cancer cell lines such as murine Neuro-2A.

## METHODS

### Material and method for cell culturing

***Materials***

Dulbecco’s modified Eagle’s medium (DMEM)/Ham’s F-12 50/50, MEM vitamins, penicillin–streptomycin and trypsin were obtained from Mediatech/Cellgro. Fetal bovine serum was obtained from Atlanta Biologicals. 75cm^2^ vented angle neck flasks were obtained from Fisher Scientific and 96-well tissue cultured-treated plates from Corning Costar.

***Cell culture***

The cells used were murine Neuro-2A (*Mus musculus*) (N2a) cells obtained from American Tissue Culture Collection (ATCC). Mouse NB was grown in DMEM/Ham’s F-12 50/50 with 1% MEM vitamins, 0.1 μg/ml penicillin, 0.1 μg/ml streptomycin and 10% heat-inactivated fetal bovine serum. The cells were grown as monolayers in 75cm^2^ vented angle neck flasks. The medium was changed every 48 h. Cells were maintained in the exponential grade phase by splitting the cells with 0.25% trypsin plus 0.1% EDTA when cells reached 90% confluence. Cells used for experiments were raised by trypsinization and diluted in medium to a density of 4.5 X10^4^ cells/ml. 100ul of diluted cells were added to each well of a 96-well tissue culture plate and tested for 24 h post-plating. The level of confluence was between 60% and 70% at the time of experimentation, with cells in the exponential grade phase. This provided a population of cells that were primarily in the G_1_ (Gap 1) or M phase (mitotic phase) of the cell cycle with very minimal differentiation which is also characteristic of aggressive human NBs. N2a cells do not display contact inhibition when grown under normal culture conditions and thus continue to divide until the cells lift once contact with the culture dish is insufficient [23]. Thus, due to the nature of the N2a cell line, which is like aggressive NB cancers, we were limited to testing cells in G_1_ or M phase of the cell cycle. It has also been shown that radiation damage to some human tumor cells inhibits the progression of G_1_ cells into the DNA synthesizing (S) phase of the cell cycle, as well as enhancing apoptosis [24]. We did not have the option of synchronizing the cells in the cell cycle or testing cells that had left the cell cycle and were in G_0_. Additionally, all cells were maintained in a humidified incubator at 37°C and 5% CO_2_.

### Laser trap set-up

**Fig. 1:**
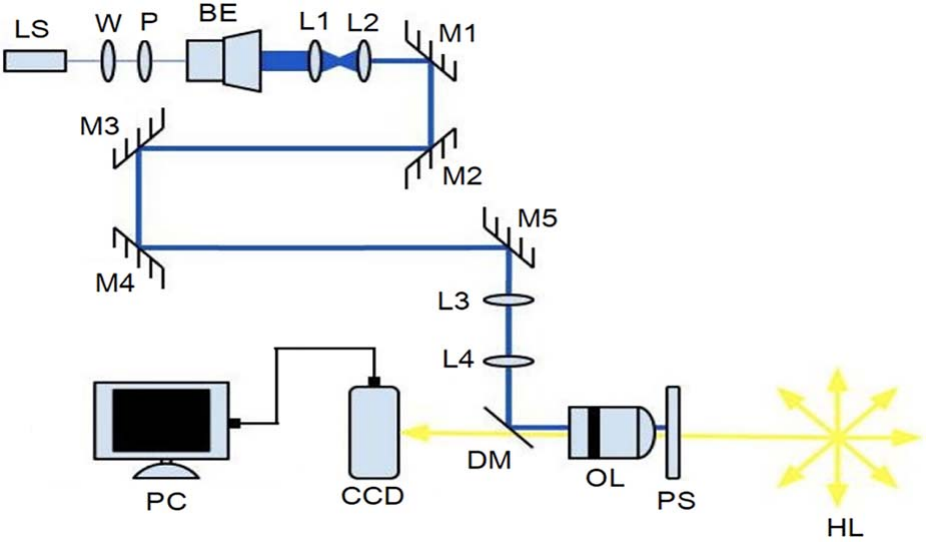
Laser trap experimental set up: laser (LS), λ/2-wave plate (W), polarizer (P), Beam Expander (BE), lenses (L_i = 1,…,4_), mirrors (M_i = 1,…,5_) dichroic mirror (DM), digital camera (CCD), optical lens (OL) and piezo-driven stage (PS) [18–22].

The essential elements of the experimental setup [18–22] used to conduct this study are shown in Fig. 1. The Laser trap experimental setup had three main parts: the high-power infrared laser, the laser beam controlling units and the inverted microscope with a computer-controlled digital camera. The high-power infrared laser (LS) was linearly polarized with an infrared diode laser which emits a maximum of 8 W power at a 1064 nm wavelength range. This high-power laser is expected to be controlled to reach the trapping point being focused on. To that end, the controlling units used half-wave plates (W) to control the power in combination with a polarizer (P), the beam expander (BE) to increase the original beam size of 4 mm by a factor of 20, two converging lenses (L1 and L2) to resize the beam to 2 cm and the optical mirrors (M1, M2, M3 and M4) for redirecting. The beam is better aligned as M5 is positioned at the proper distance. This creates a steerable trap in the microscope’s focal plane. M5 is positioned at 20 cm, the focal length of the converging lens (L3) and 60 cm from the converging lens (L4), such that the distance between L3 and L4 is twice their focal length. L4 is situated 20 cm from the back of the objective lens. This setup creates the steerable trap on the microscope’s focal plane.

Then, the collimated and aligned beam with a power of (~4.36 W) was directed to the inverted microscope and the beam power, after passing through the slide holding the sample, was ~0.839 W with a deduced efficiency of about ~19.24%. At the same time, the DM transmitted the imaging light from the Olympus Tl4 halogen lamp for the live image captured by a PC-controlled digital camera integrated into the microscope.

### Experimental procedure

**Fig. 2:**
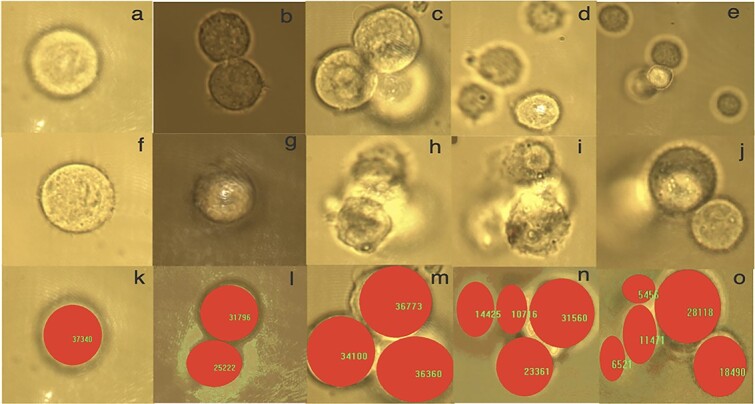
Single and Mass cell ionization: First row describes the images captured as the cells being attracted to the trap. The second row describes the images captured after all cells are inside the trap. The third row describes how the area of the cells inside the trap is measured (showing the number of pixels). Each row from left to right represents one, two, three, four, and five cells. The images taken for NB cells before (first row) and after trapping (second row): single-cell (a, f); 2-cells (b, g); 3-cells (c, h); 4-cells (d, i); 5-cells (e, j). In the third row (k-o) ImageProplus6 was used to measure (in pixel) the areas of one to five cells.

The N2a sample was sucked by a micro-pipet and deposited on a dispersion slide, covered with a coverslip and then mounted on the piezo-driven stage (PS) of the microscope. The cells lying on the bottom of the slide would often quickly stick to the coverslip of the slide which required tapping to separate it for trapping. A single cell was trapped using the high-intensity gradient infrared laser at 1064 nm. It became ionized after some instant of time. Similarly, 2-cells, 3-cells, 4-cells and 5-cells were trapped and ionized for a total of 60 cells for each group created (1–5 cells). We shot consecutive images of the cell before it was trapped using a digital camera. We then opened the gate at the microscope’s laser port, allowing the cell to become instantly trapped and ionized. Real live successive images illustrating this process are shown in Fig. 2 for each of the five individually ionized cell clusters (single-cell, two- cells, three-cells, four cells, and five cells ionization). The cell size is determined by counting the number of pixels in each cell for 1-cell - to 5-cells (see Fig. 2 (k) to (o)); these pixels are then converted to meters to determine mass of the cells or cell cluster.

## THEORY OF CELL IONIZATION

### Cell-membrane dielectric breakdown

The cell membrane protects the cell from its environment and regulates everything that enters and leaves the cell. This biological cell membrane is very thin (about 5–10 nm thick) [25] and composed of a lipid bilayer into which proteins are embedded. Within this small distance, the membrane allows the water dissolved ions [26], to surround the bilayer on each side of the membrane creating a membrane potential. Thus, an electrostatic field exists across the cell membrane. Since the ions cannot move across the membrane, the lipid membrane acts as a capacitor, and proteins in the bilayer behave as a dielectric constant of approximately 2–4 [27–29], betraying the existence of the electric field crossing the lipid membrane. If the ions are moved from one side of the membrane to another side, they generate an electric field with a potential difference across the membranes called the membrane potential [Stipendnew-Certification-and-Payment-Request]. Therefore, the lipid membrane can be considered an electrical capacitor. A potential difference is set in the lipid membrane due to the separation of charges at the opposite end. This induced potential causes the cell membrane to become highly conductive or extremely polarized, resulting in the induced dipoles being misaligned and creating a torque [19].

However, suppose the exposure is to a strong and rapidly oscillating electric field (such as that generated by the laser field). In that case, the membrane does not reseal, and this leads to irreversible electroporation. The electrons are permanently separated from the atoms which causes the cell to ionize. The charges build up gradually, increasing the electric force which results in the total dielectric breakdown as this force is larger than the gradient trapping force. As a result, the N2a charge cell is ejected. The amount of the radiation energy of the laser incident absorbed by the cell determines the TIE needed for cell death from the time the cell enters to it leaving the trap.

### TIE and radiation dose

N2a cell samples were placed on a well slide, that was then mounted on the microscope stage. Each N2a cell was radiated by a high gradient electric field of the laser, and they gradually built a greater electrostatic force that overcame the trapping force within the cells; as a result, they ejected from the trap and disappeared from the camera’s view range. While this phenomenon is unfolding, the TIE needed to kill the cancer cell is determined by the amount of radiation energy that is absorbed by the cell. Therefore, our study mainly focused on the radiation response of a single and multi-N2a cell by computing and analyzing the TRD that hinges on the equivalent mass and the average radiation energy captured by each cell. To find out the basic physical quantities that would express the cell’s biophysical properties, a spherical model for N2a cells were considered and the diameter (D) of each cell was determined from the image of each cell using the software ImageProplus6 in pixels with a conversion factor of $7.27\times{10}^{-8}$  *meter/pixel* before it was trapped (see Fig 2 (k–o)). This was found by using a 3.1 μm silicon bead’s diameter. The mass of each cell was calculated by assuming the density of cancer cells is that of water [30, 31]:

(1) \begin{equation*} {\mathbf{M}}_{\mathbf{Cell}}=\frac{\boldsymbol{\pi}}{\mathbf{6}}\boldsymbol{\rho}\ {{\mathbf{D}}^{\mathbf{3}}}_{\mathbf{cell}} \end{equation*}

The average power incident on the cell, which was kept the same throughout the ionization of each cell, was P_I_ = 0.839 W and the average estimated transmitted power was, P_T_ = 0.69 W. Using the area ratios (from cell and beam area), the mass of the cell, the absorbed power of the cell (difference in incident & transmitted power), and the time that the cell was trapped, the average TIE for each cell could be determined:

(2) \begin{equation*} \mathbf{TIE}=\frac{{\mathbf{A}}_{\mathbf{cell}}}{{\mathbf{A}}_{\mathbf{beam}}}\left[{\mathbf{P}}_{\mathbf{I}}-{\mathbf{P}}_{\mathbf{t}}\right]\times{\mathbf{T}}_{\mathbf{I}} \end{equation*}

T_I_ determines the ionization period which is the time between when each cell entered the trap until it leaves the trap. As in our previous work [32], we estimate beam size as follows:

(3) \begin{equation*} {\mathrm{A}}_{\mathrm{beam}}=\pi{\omega}_0^2. \end{equation*}

where ${\omega}_o$is beam waist distance. TRD was determined using the mass calculated for each cell from equation (1) and the average radiation energy absorbed in equation (2).

(4) \begin{equation*} TRD=\frac{TIE}{M_{cell}}. \end{equation*}

## RESULTS AND DISCUSSION

As explained in the above sections, this study presents a systematic investigation of TIE and Radiation Dose vs. Mass for both single and multi-cell ionization processes, using LT techniques for N2a cells. The experimental results are presented and discussed for single cell, 2-cells, 3-cells, 4-cells, and 5-cells with a general comparison of each N2a- cells ionization in the following sections. To establish a clear relationship between the TIE and TRD with the mass of the cells, we have made statistically valid data reduction by using Origin 2019 a graphical data analysis program software. In this statistical reduction method, we first sorted each data by the TIE or TRD in ascending order and eliminated the two maximum values from each. A second sorting was performed by mass in ascending order, again eliminating the two maximum values. A further reduction was carried out by subgrouping the data with mass increment and calculating the average mass for TIE and TRD. The result is displayed graphically for single cell, 2-cells, 3-cells, 4-cells, and 5-cells.

### Single cell ionization of N2a

A single cell ionization is a process of trapping one cell by a high-power infrared laser beam to build its charge until fully ionized. Based on the experimental procedures, a total of 60 NB cells for a single cell ionization process were studied. For these cells, the basic statistical parameters for the average cross-sectional area (A_cell_), Diameter (D_cell_), volume (V_cell_), mass (M_cell_), ionization-time (T_I_), TIE and TRD are measured and calculated (see Table 1).

**Table 1:** Basic statistical parameters measured and calculated: Area, Mass, TIE and TRD for N2a Cells

**Quantities**	**Mean**	**SD**	**Min.**	**Med.**	**Max.**
**Single Cell ionization**					
A_Cell_ (μm^2^)	159.4	35.8	105.2	155.7	250.2
D_Cell_(μm)	14.2	1.6	11.6	14.1	17.9
V_Cell_(μm^3^)	1542.4	517.9	811.7	1462.2	2977.6
M_Cell_(ng)	1.5	0.5	0.8	1.4	3.0
T_I_ (sec)	181.2	40.7	112.0	179.0	287.0
TIE (mJ)	61.8	13.9	38.2	61.1	97.9
TRD (J/μg)	42.6	11.5	23.2	39.7	81.4

By using the area ratios (the laser beam (A) area and the cell (ACell) area), the mass of the cell (MCell), the absorbed power of the cell, and the time spent in the trap, the total IE for each cell was calculated. According to the findings (Table 1), TIE varies from a minimal of 38.2 mJ to a 97.9 mJ maximal with an average of 61.8 *± 13.9 mJ*. Similarly, TRD value was analyzed and varied from a minimal of 23.2 J/μg to a maximal 81.4 J/μg with an average of $42.6\pm 11.5\ \mathrm{J}/\mu \mathrm{g}$.

**Fig. 3:**
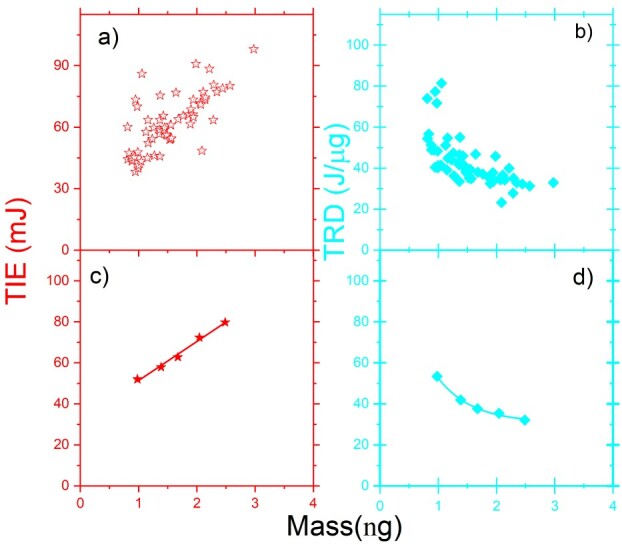
The TIE (a–c) ($ \color{red}{\textrm{red}}$) and TRD (b–d) ($ \color{cyan}{\textrm{cyan}}$) versus mass for a single N2a cell: (a) and (b) for all cells; (c) and (d) for the reduced data.

To predict the relationship between the TIE and the mass of a single trapped N2a cell, we analyzed the TIE and TRD as a function of the mass of the individual cells. Fig. 3(a–c) displays the TIE versus mass ($ \color{red}{\textrm{red}}$), and Fig. 3(b–d) indicates TRD versus the mass ($ \color{cyan}{\textrm{cyan}}$) of N2a cells for a single trapped cell. The reduced data is shown in Fig. 3(c) for the TIE and in Fig. 3(d) for the TRD. These results show that TIE increases with the mass of cells, while an inverse relationship between the TRD and the mass of the N2a cells in a single cell ionization process is observed.

### Double-cell ionization of N2a

The process for two ionized N2a cells was carried out for a total of 60 cells successfully. During these cells’ ionization process, only two cells were ionized per slide. This was done to reduce the electrostatic interaction between the ionized cells and temperature to due IR ionization radiation. When trapping the other cells around them, a safeguard was taken to move in the direction away from the recently ionized two cells. The basic statistical parameters can be analyzed and tabulated in Table 2. From the findings (Table 2), the TIE varies from a minimal of 26.7 mJ to a maximum of 138.9 mJ with an average of 64$.3\pm 28.7\ mJ$ whereas the TRD varies from a minimal of 10.4 J/μg to a maximal 32.7 J/μg with an average of$19.8\pm 5.1\ \mathrm{J}/\mu \mathrm{g}$. The TIE and TRD as a function of the mass of individual cells were analyzed.

**Table 2:** Values of essential statistical parameters for measured and calculated N2a cells with laser trap

**Quantities**	**Mean**	**SD**	**Min.**	**Med.**	**Max.**
**Double Cell ionization**					
A_Cell_ (μm^2^)	269.6	101.2	118.6	244.4	657.2
D_Cell_(μm)	18.2	3.2	12.3	17.6	28.9
V_Cell_(μm^3^)	3491.1	2141.7	971.4	2873.4	12673.3
M_Cell_(ng)	3.5	1.3	0.6	1.8	7.9
T_I_ (sec)	264.8	118.2	110.0	230.0	572.0
TIE (mJ)	64.3	28.7	26.7	55.9	138.9
TRD (J/μg)	19.8	5.1	10.4	19.1	32.7

**Fig. 4:**
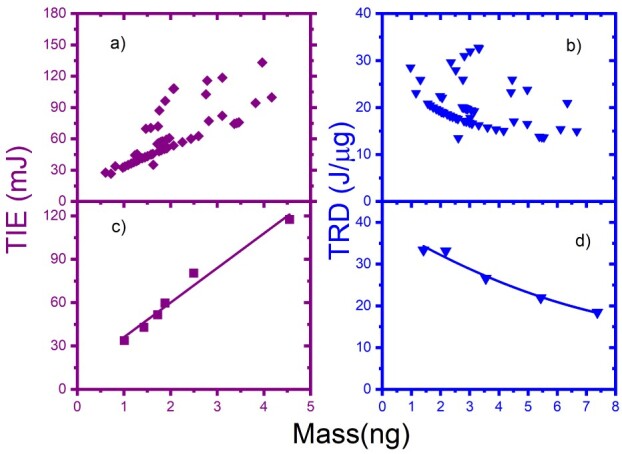
The TIE (a–c) in purple and TRD (b–d) in blue vs. mass for the 2-trapped cells of an N2a cancer: (a) and (b) for all cells; (c) and (d) for the reduced data; (c) and (d) data are curve fitted.

The results in Fig. 4(a–d) show the TIE (**purple**) and TRD (**blue**) versus the mass of NB cells for double trapped cells. The reduced data is shown in Fig. 4(c) for TIE and Fig. 4(d) for TRD. Additionally, the reduction was performed by subgrouping the data in Fig. 4(c–d) for TIE and TRD. Both graph results indicate as TIE increased and TRD decreased when the values of the mass increased.

### Triple-cell ionization of N2a

The ionization of the trapped three-cell sets was difficult because of their entry time variation. This is caused by the other cells moving around them. The result is that the cells enter at different times but are ejected from the trap simultaneously after ionization. This allows the ionization period to be known fairly well. Using the same procedure as single-cell ionization, a total of 60 cells analyses were also carried out. The basic statistical parameters are given in Table 3. From these parametric values, we found that the TIE obtained varies from a minimal of 31.2 mJ to a maximum of 113.8 mJ with an average of 73$.\mathbf{1}\pm \mathbf{19.7}\ \mathbf{mJ}$. Using equation 4, the TRD was calculated, and its result varied from a minimal of 4.9 J/μg to 24.1 J/μg with an average of $\mathbf{12.7}\pm \mathbf{3.2}\ \mathbf{J}/\boldsymbol{\mu} \mathbf{g}$.

**Table 3:** The values of essential statistical parameters of the three-trapped and ionized N2a cells with a laser trap

**Quantities**	**Mean**	**SD**	**Min.**	**Med.**	**Max.**
**Triple Cell ionization**					
A_Cell_ (μm^2^)	419.6	120.2	200.8	388.7	799.0
D_Cell_(μm)	22.8	3.2	15.9	22.2	31.9
V_Cell_(μm^3^)	6657.6	2918.8	2141.0	5764.9	16990.4
M_Cell_(ng)	6.7	2.9	2.1	5.8	16.9
T_I_ (sec)	383.4	102.7	163.0	358.0	594.0
TIE (mJ)	73.1	19.7	31.2	68.6	113.8
TRD (J/μg)	12.7	3.2	4.9	11.9	24.1

**Fig. 5:**
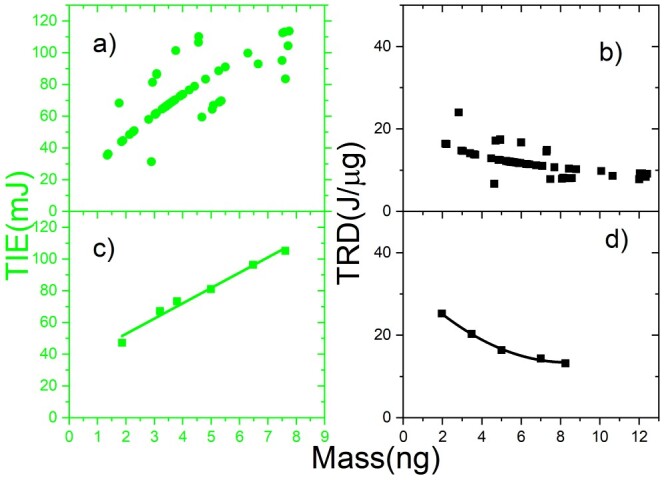
The TIE (a–c) in green and TRD (b–d) in black vs mass for the 3-trapped cells of a N2a cancer: (a) and (b) for all cells; (c) and (d) for the reduced data.

In an attempt to study the connection between the radiation IE, and the mass of the N2a cells, we have examined the TIE and TRD as a function of the mass of the individual cells. The results in Fig. 5(a–d) display the TIE ($ \color{green}{\textrm{green}}$) and TRD (**black**) vs mass for all NB-cells. The reduced data is shown in Fig. 5(c) for TIE and Fig. 5(d) for TRD. The reduction was made by subgrouping the data with mass increment and calculating the average mass for TIE and TRD. The results of both TIE and TRD versus mass graphs show increasing values as mass increases and decreases in TRD as mass increases.

### Four-cell ionization of N2a

In this ionization process, a total of 60 N2a cells were studied in the form of multi-cell ionization which means 4-cells in a trap were ionized using the high gradient laser beam. The basic statistical parameters are given in Table 4. The TIE varies from a minimum of 43.2 mJ to a maximum of 166.4 mJ with an average of$\mathbf{83.2}\pm \mathbf{26.6}\ \mathbf{mJ}$ and TRD vary from a minimum of 6.2 J/μg to 14.2 J/μg with an average of $\mathbf{9.5}\pm \mathbf{1.6}\ \mathbf{J}/\boldsymbol{\mu} \mathbf{g}$. The TIE and TRD as a function of the mass of individual cells were analyzed.

**Table 4:** The values of essential statistical parameters N2a of the four-trapped and ionized N2a cells with a laser trap

**Quantities**	**Mean**	**SD**	**Min.**	**Med.**	**Max.**
**Four Cell ionization**					
A_Cell_ (μm^2^)	527.2	170.3	281.2	480.5	1082.2
D_Cell_(μm)	25.6	3.9	18.9	24.7	37.1
V_Cell_(μm^3^)	9440.4	4805.4	3547.4	7924.1	26782.7
M_Cell_(ng)	9.4	4.8	3.5	7.9	26.8
T_I_ (sec)	491.1	157.9	255.0	443.0	982.0
TIE (mJ)	83.2	26.6	43.2	75.1	166.4
TRD (J/μg)	9.5	1.6	6.2	9.5	14.2

**Fig. 6:**
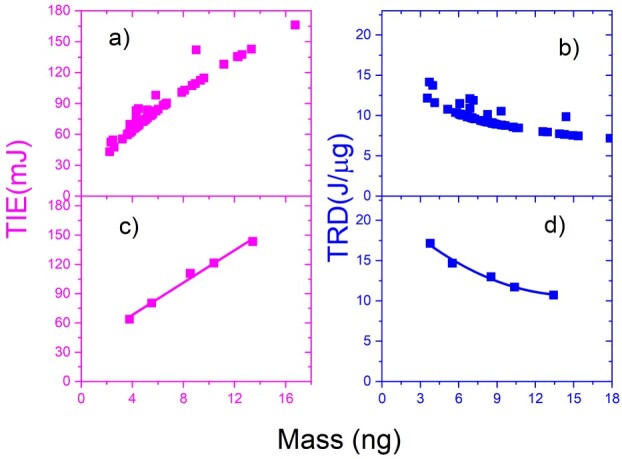
The TIE (a–c) in magenta and TRD (b–d) in blue vs mass for the 4-trapped cells of N2a cancer: (a) and (b) for all cells; (c) and (d) for the reduced data.

The results in Fig. 6(a–d) display the TIE (magenta) and TRD (blue) versus the mass of NB cell for four trapped cells. The reduced data is shown in Fig. 6(c) for TIE and Fig. 6(d) for TRD and the reduction was made by subgrouping the data and calculating the average mass for TIE and TRD. The results of both TIE and TRD versus mass graphs show increasing values as mass increases and decreases in TRD as mass increases.

### Five-cell ionization of N2a

In the same procedure as 2-, 3- and 4-cell trapping techniques, we have made the 5-cell ionizing method in a trap. A total of 52 cells were carried out, and the basic statistical parameters were studied and given in Table 5. From these parametric values, the TIE varies from a minimal of 44.8 mJ to a maximal of 153.6 mJ with an average of $\mathbf{88.2}\pm \mathbf{23.6}\ \mathbf{mJ}$ whereas the TRD value varies from a minimal of 5.6 J/μg to a maximal of 8.3 J/μg with average values of $\mathbf{6.9}\pm \mathbf{0.6}$ J/μg. The TIE and TRD as a function of the mass of individual cells were analyzed.

**Table 5:** The values of essential statistical parameters of the five-trapped and ionized cells with a laser trap

**Quantities**	**Mean**	**SD**	**Min.**	**Med.**	**Max.**
**Five Cell ionization**					
A_Cell_ (μm^2^)	664.5	157.5	413.2	638.6	1095.7
D_Cell_(μm)	28.9	3.4	22.9	28.5	37.4
V_Cell_ (μm^3^)	13146.5	4784.3	6318.9	12138.4	27283.6
M_Cell_ (ng)	13.1	4.8	6.3	12.1	27.3
T_I_ (sec)	539.2	144.5	274.0	521.0	939.0
TIE (mJ)	88.2	23.6	44.8	85.2	153.6
TRD (J/μg)	6.9	0.6	5.6	7.0	8.3

**Fig. 7:**
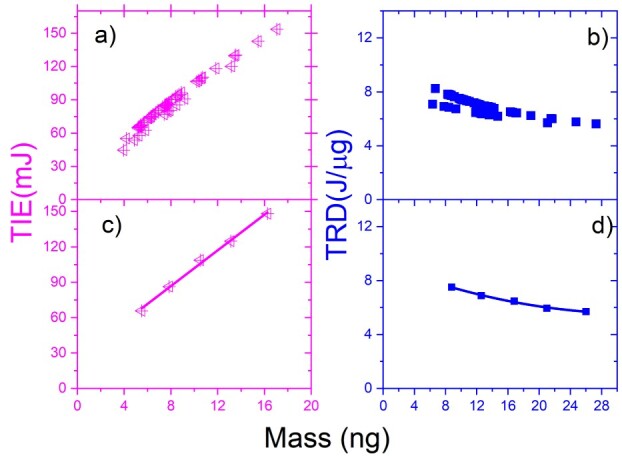
The TIE (a–c) in magenta and TRD (b–d) in blue vs mass for the 5-trapped cells of an N2a cancer: (a) and (b) for all cells; (c) and (d) for the reduced data and fitted.

The results in Fig. 7(a–d) display the TIE ($ \color{magenta}{\textrm{magenta}}$) and TRD ($ \color{blue}{\textrm{blue}}$) vs the mass of N2a cell for a five trapped cell. The reduced data is shown in Fig. 7(c) for TIE and in Fig. 7(d) for TRD, and the reduction was made by subgrouping the data and computing the average mass for TIE and TRD. The results of both TIE and TRD versus mass graphs show an increase in values as mass increases and a decrease in TRD as mass increases.

### General comparisons of each N2a cells ionization

In the previous sections, a single and multiple cells ionization (2-5) of N2a cells’ TIE and TRD have been discussed. In the TIE and TRD studies for each N2a cell, during the trapping process, we had to keep each trapped cell isolated from the other cells until they were fully ionized and ejected from the trap. In this time frame, the gradient force attracted adjacent cells, which jumped into the trap at different times. It resulted in capturing multiple cells in the trap. Regardless of the timing of cells entering the trap, all trapped cells were expelled simultaneously at the end of ionization. Individual N2a cells in a trap spend relatively more time in the trap as the number of cells increases. Moreover, the ionization period of a single cell of N2a was longer as compared to other small cell studies [18], and shorter than 4T1 cancer cells [22] due to cell size difference.

In order to compare the energy (TIE) and radiation dose (TRD) of N2a cells, we created five groups built on the number of cells that were in the trap for the duration of the ionization period: 1-cell, 2-cells, 3-cells, 4-cells, and 5-cells. The aim of this inquiry is to see how the TIE and TRD are impacted by the accumulation of one or more cells while the first cell goes undergoes membrane breakdown and charges continue to accumulate due to radiation damage.

**Fig. 8:**
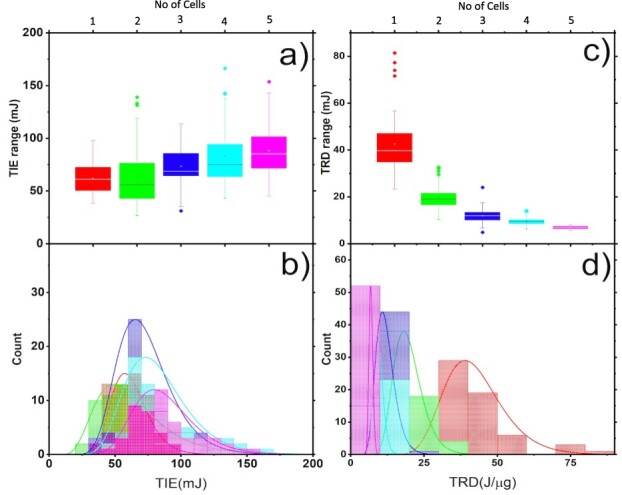
The statistical distributions for the TIE (a–b) and the TRD (c,d) for single ($ \color{red}{\textrm{red}}$), double ($ \color{green}{\textrm{green}}$), triple ($ \color{blue}{\textrm{blue}}$), four ($ \color{cyan}{\textrm{cyan}}$) and five ($ \color{magenta}{\textrm{Magenta}}$) NB cells.

As the results indicated in Table 6 show, the mean values of absorbed TIE, for every cell (from 1-cell to 5-cells), were found to be TIE = 61.8 ± 13.9 mJ, 64.3 ± 28.7 mJ, 73.1 ± 19.7 mJ, 83.2 ± 26.6 mJ and 88.2 ± 23.6 mJ respectively. The higher dispersion of a set of values from these results is caused by the wide range of cell sizes which affected the magnitude of the IE. The findings for the TIE and TRD for multiple cells alongside single ionization are displayed in the box-and-whisker plot in Fig. 8.

**Table 6:** The values of essential statistical parameters of all trapped and ionized N2a cells with a laser trap

N2a-Cells													
Subgroup	N	TIE (mJ)				Mass (ng)				TRD (J/$\mu$g)			
		Mean	SD	Min	Max	Mean	SD	Min	Max	Mean	SD	Min	Max
1	60	61.8	13.9	38.2	97.9	1.5	0.5	0.8	3.0	42.6	11.5	23.2	81.4
2	60	64.3	28.7	26.7	138.9	3.5	1.3	0.6	7.9	19.8	5.1	10.4	32.7
3	60	73.1	19.7	31.2	113.8	6.7	2.9	2.1	16.9	12.7	3.2	4.9	24.1
4	60	83.2	26.6	43.2	166.4	9.4	4.8	3.5	26.8	9.5	1.6	6.2	14.2
5	52	88.2	23.6	44.8	153.6	13.1	4.8	6.3	27.3	6.9	0.6	5.6	8.3

The left histograms in Fig. 8 are for the TIE and the right side for the TRD. In each of the histograms, the 1-cell, 2-cells, 3-cells, 4-cells, and 5-cell traps are represented by red, green, blue, cyan, and magenta respectively. From the distribution curves on the lower panel Fig. 8(b), the shift within the peak values indicates that the TIE increases with the number of cells within the trap (Fig. 8(a)). Moreover, TRD approaches a minimum value with increased mass, and as the number of cells increases in the trap, this value decreases (see Figs 3–7).

The summary of the essential statistical parameters for the TIE is shown in Table 6. The outcomes clearly indicate that the average TIE for the five groups (1-cell-5-cells) increases because the number of cells increases consistently. A similar quantitative comparison was carried out within the groups (1–5 cells) by analyzing the relative TIE percentage increase, as was done in the case of 4TI breast carcinoma cells [22].

With the field inactive, theoretically, there should be a 200–500% relative increase in the TIE for the 2–5 cells compared to a single cell for any of the five groups with computed values of 3.9%, 15.5%, 25.7%, and 29.9%. This is due to the internal electrical effects in cells caused by the infrared laser affecting the estimation of TIE in multiple cells [19]. There is a similar TRD for single and multiple cells based on the same graphs, and because of this, the colors on the respective sides of Fig. 8 are identical. In contrast to the TIE, the distribution curves of the five groups (Fig. 8(d)) taken in total indicate a transferal to the left, which shows a reduction in TRD with a rise in the number of cells within the trap. This shift is also displayed in Fig. 8(d). Table 6 presents the essential statistics of the TRD.

Based on the results, the mean value of the TRD decreases steadily: 42.6 – 6.9 J/μg. The relative decrease in multiple (2–5) cells’ average TRD as compared to the single cell was found to be 53.5%, 70.2%, 77.7%, and 83.8%. We also compared the relative TRD between consecutive cells, which was found to be 53.5%, 35.9%, 25.2%, and 27.4%. This decrease shows that it takes less energy to ionize multiple cells than a single cell. The results for multiple cell ionization in both TIE and TRD are significant; they compare to single cell results in terms of predicting physical processes.

To understand what is observed, we employed a similar diminution process studying how TIE and TRD evolve with the mass of an increasing number of cells entering the trap (see Fig. 8). The figures show TIE on the left axis and TRD on the right following the code: (single cell (red), (two cells (green), three cells (blue), four cells (Cyan), and five cells (Magenta)). Main TIE and TRD vs mass calculations are indicated in the row below (b) and (d), while those of (a) are in the top row, and (c) shows the linear fit data reduced as seen in section 4.1. Fig. 8(a, c) single cell reduced data shows an elevation of multiple cell ionization mass for TIE and TRD. The continued cell charging, and temperature increase from the infrared caused multiple cell ionization to reduce TRD.

The process of irradiation damages DNA, which kills cancer cells. We know that the lowest deliverable energy (TIE) and radiation (TRD) are altered [18]. We can model an N2a cell as a sphere with several dielectric dipoles when there is no radiation. In the presence of radiation, on the other hand, its fast oscillating laser radiation field causes the dipoles to exhibit altered behaviors as they increase oscillation to line up with the field’s polarization.

In the absence of the electric field, there is no global polarization, and this results in a stronger oscillation within the cell. The weakest dipoles will be disrupted by the excessive electrical field. Free charges form inside the cell when the electrical field becomes strong (high power radiation). Due to the fact that the cell is composed of different sorts of molecules with different dipole strengths, the dipoles break at different times and need different amounts of energy. This may result in gradual charging that occurs with time. The beam produces varying field strengths in the cell, depending on where it is pointed. In addition to contributing to the gradual charge buildup inside the cell, the gradient contributes to the stability of the cell. Multiple cells entering a trap at different times could significantly raise the edge radiation dose. Due to the breakdown of the dielectric by the radiation on the primary trapped cell, the charge continues to build up; as the rest of the cells enter the trap, they are impacted by the electrical field of the trapped cell, combined with the rapidly oscillating radiation field. An extra effect of ionization can be attributed to free charges in the biological cell(s) that is (are) already in the trap. As a result, TRD decreases as the number of cells increases.

### The comparison between N2a and 4T1 cells lines

**Fig. 9:**
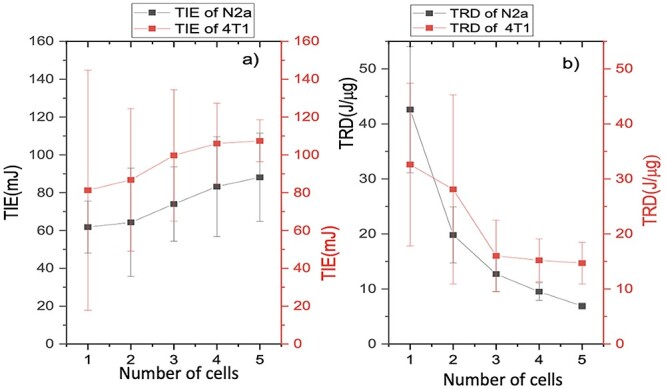
Comparison plot of TIE and TRD vs the number of N2a and 4 T1 cells, (a) TIE for N2a and 4 T1 (b) TRD for N2a and 4 T1.

Fig. 9 illustrates the results of the two experiments on N2a (**black**) and 4T1 ($ \color{red}{\textrm{red}}$) cancer cells TIE and TRD values vary with the number of cells in the trap. Our findings for TIE and TRD were similar to the previous result [22]. TIE increased with the cell number in both N2a and 4T1. N2a cells are on average smaller than 4T1 cells, which explains the difference in the TIE energy. The trend in TRD is comparatively similar for N2a and 4T1 cells (see Fig. 9(b)). Based on mice’s origins, the N2a cell line has both neuronal and amoebic stem cell characteristics, allowing it to respond to environmental influences. Numerous neuronal features, including neurofilaments [33] can be found in this cell type. Tumorigenicity and invasiveness of the 4T1 cell line are higher [34].

Let us recall that in RT, the therapeutic ratio is the maximum radiation dose by which the death of cancer cells is locally controlled and the minimum radiation dose by which cells in normal tissues have low acute and late morbidity [35]. Therefore, since TRD is decreased with the increased mass of N2a and 4T1 cells, we believe that the chain ionization mechanism [20] brings about this reduction in TRD (Fig. 9(b)). Using single-cell ionization, we are able to determine the precise amount of energy to kill target cells. RT is effective as a result that normal cells do not lose the DNA repair mechanism as compared to most cancer cells that have deficiencies in DNA repair factors [13, 36–38]. Thus, the determination of the single cell IE is the maximum energy that is required for safe radiation dosing. We can speculate that since a tumor is a cluster of single cells, our results are demonstrating that by knowing the maximum energy required for mitotic catastrophe/necrosis, we can adjust the amount of energy required to destroy a tumor based on size and mass.

We can conclude that a therapeutic ratio decreases as a result of the chain ionization effect occurring in the presence of multiple cells. Looking at Fig. 9(b), we can also reason that since TRD decreases with the increase of cell mass for both N2a and 4T1, then there is a way forward in RT for better and more precise treatment plans. Additionally, since our method allows for a very precise way of determining radiation energy, then the potential for a better therapeutic ratio based at the cellular level to reduce radiation-induced tissue toxicity while advancing the sterilization of the cancerous cells is demonstrated here.

## CONCLUSION

We have presented a single and mass cell ionization of NB carcinoma cells. These ionization processes were studied by measuring and comparing the TIE and TRD of N2a cancer cell sample, which could be important in the treatment of NB in children. The TIE and TRD were found through a new approach that uses LT techniques for single and multiple cell ionization. The results obtained from a single cell were analyzed by 2D scattering graphs which show that TIE increased as the mass of each individual cell increased while the TRD decreased as the mass of the cells increased. This clearly validates the effectiveness of RT of NB cells. Most notable is the fact that the TRD phenomenon observed is contingent on and strongly connected to mass, especially in the ionization of multiple cells (2–5 cells). Based on the result observed, we can conclude that multiple cells’ TIE and TRD vs mass induced charge are very important. We observed that TRD decreased as the mass of cells increased. This diminution in TRD gets to be more significant in multiple cell ionization as we see in TRD vs the number of cells entering the trap. The significant drop in multiple cell ionization TRD is related to the ionization domino effect caused by the radiation field and the absorption by water molecules at 1064 nm. In light of our results here, it is possible to develop a computational model that predicts TRD for *in vivo* treatment by generalizing multiple cell ionization and radiation dose data derived from the results here with more precise information about tumor size and density.

Nonetheless, further research is needed that all combine pre/during/post ionization dynamics in multiple and single cell ionization along with the dosage and period of radiation treatment. One such critical follow-up study should focus on the exact estimation of the temperature and charge rise as radiation meets the cell(s).

## CONFLICT OF INTEREST

The authors have no conflicts of interest to declare. All authors have seen and agree with the content of the article and there is no financial interest to report.
